# Manipulating guided wave radiation with integrated geometric metasurface

**DOI:** 10.1515/nanoph-2021-0466

**Published:** 2021-10-13

**Authors:** Bin Fang, Zhizhang Wang, Shenglun Gao, Shining Zhu, Tao Li

**Affiliations:** National Laboratory of Solid State Microstructures, Key Laboratory of Intelligent Optical Sensing and Manipulation, Jiangsu Key Laboratory of Artificial Functional Materials, College of Engineering and Applied Sciences, Nanjing University, Nanjing 210093, China; Collaborative Innovation Center of Advanced Microstructures, Nanjing 210093, China; College of Optical and Electronic Technology, China Jiliang University, Hangzhou 310018, China

**Keywords:** guided wave, metasurface, optical field manipulation, thin-film lithium niobate

## Abstract

Metasurfaces have manifested unprecedented capabilities in manipulating light by subwavelength unit cells, facilitating the miniaturization and multifunctions of optical systems. On the other hand, lithium niobate on insulator (LNOI) technology is revolutionizing the integrated photonics, enabling multifunctional devices and applications. Yet the optical interface for coupling and manipulation is not sufficient and versatile. Here, we developed a geometric metasurface interface for LNOI waveguide and demonstrated several on-chip integrated devices for free space light field manipulations. By decorating waveguides with subwavelength optical antennas, we manipulated the guided waves into desired wavefronts in space, achieved complex free-space functions, such as focusing, multichannel vortex beam generation, and holography. Our architecture goes beyond the conventional gratings and enriches the functionalities of metasurface, which would open up a new perspective for future versatile guided-wave driven optical devices.

## 1 Introduction

The development of integrated photonics empowers people to integrate light sources, modulators, optical antennas, and other functional components in a tiny chip for in-plane light routing and out-of-plane radiation. As a traditional optoelectronic material, lithium niobate (LN), acclaimed as “the Silicon of Photonics” [1], has been widely investigated for its excellent properties, including relatively high refractive index, a wide optical transparency window, large electro-optic coefficient as well as large second-order nonlinear susceptibility [2]. Especially after the birth of lithium niobate on insulator (LNOI), there has been encouraging progresses in developing diverse thin-film LN devices for photonic integrations [3], [4], [5], [6], [7], [8], [9]. To take full advantages of LN waveguides in free-space radiation applications, it is important to have an interface that can flexibly manipulate light when converting between guided wave and radiation modes. Nevertheless, traditional interfaces, like surface gratings [10] and edge couplers [11], have limited functionality and lack complete control of light. Grating arrays is an alternative interface which can perform more advanced functions, such as off-chip beam deflection [12], focusing [13], and holography [14], while it suffers from high-order diffraction loss as well as large footprints.

On the other hand, metasurface as newly-emerging ultrathin design is highly expected to revolutionize optical devices and technology. Through well-designed artificial nanostructures, light can be flexibly manipulated with multiple degrees of freedom like amplitude, phase, polarization, and enabling the miniaturization of optical systems [15], [16], [17], [18]. Although compact size is one of the major advantages of metasurfaces, most designs are based on free space illumination for miscellaneous functions, including wavefront shaping [19], metalens imaging [20], and holograms generation [21], which makes it difficult to implement full on-chip integration. Very few works are reported about the compact integration that breaks conventional optical settings, e.g., a metalens-based microscope [22]. Nevertheless, integrating metasurface to waveguides would be a highly-valuable scenario, including spatial optical coupling [23], polarization sorting [24, 25], mode routing [26, 27], mode conversion [28, 29] and frequency conversion [30, 31]. Particularly, Ni et al. demonstrated a hybrid metasurface directly driven by guided wave to realize different free space functions based on the silicon waveguide [32]. However, to cover 2*π* phase shift range imposed by the resonant principle, the parameters of the antennas are usually challenging with relatively low tolerance to the fabrication errors. The resonant phase response is also wavelength dependent, which prohibits it to work in broadband. Moreover, it is quite difficult to maintain uniform amplitude of the extracted waves meanwhile satisfy the phase condition. In view of the limitations in present designs and demands for LN integrated optical devices, a more convenient, robust and efficient interface across waveguide and free space may be worthy of attention.

In this work, we developed an integrated geometric metasurface in LNOI platform as the interface between the guided wave and free-space radiation light with versatile functions. Subwavelength antennas with different rotation angle are patterned on the top surface of the LNOI waveguide, and the guided waves are coupled out with circular polarization and manipulated into desired free-space light according to the geometric phase design. We experimentally fabricated geometric silicon (Si) metasurfaces on the LN slab waveguide by electron beam lithography and reactive ion etching process, and then demonstrated functions of integrated metalens focusing, multichannel vortex beam generation, and holographic imaging. Our approach not only breaks the limit of traditional gratings for guided wave couplers, but also extends the guided wave based geometric metasurface, which show apparent advantages compared with the commonly used resonant metasurfaces.

## 2 Fundamentals and design principles

Geometric metasurfaces, based on the geometric phase or Pancharatnam–Berry (PB) phase design, have attracted intense attention for their unparalleled capabilities in controlling the circular polarized (CP) light. Considering two identical scatters with rotation difference of an angle *φ*, if illuminated by the same CP spatial light, one can find that the spin-flipped components of scattered waves differ only with a phase factor e^i2*σφ*^, where *σ* = ±1 correspond to the helicity of right- (RCP) and left-circularly polarized (LCP) incident light. Such an abrupt phase shift is independent of the scatter details and the frequency, therefore it is a robust manipulation. For more detailed interpretation on its working principle, one may refer to some early works designed by geometric metasurface, e.g., Huang et al. [33].

It is relatively simple to construct a geometric metasurface in free space by arranging the unit cells orientation according the required phase distribution. However, the circumstance in waveguides becomes quite different. Light travels in waveguides with (quasi) transverse-electric (TE) modes or (quasi) transverse-magnetic (TM) modes and we can hardly reconfigure it into CP light with extra devices just like the free space. One feasible method is to synthetize the polarization by interference of the in-plane field. For example, if two TE guided waves propagate orthogonally (along *x* and *y* direction individually) in a slab waveguide, different local phase lags of the two modes are reconfigured when they are superposed at different positions of (*x*, *y*) as Δ*φ* = Δ*φ*_0_ + *β*(*x* − *y*), where Δ*φ*_0_ is the initial phase lag of two excited wave between the *x* and *y* directions, *β* is the propagation constant. The transverse components of the electric field (the in-plane field, *E*_*x*_ and *E*_*y*_) are orthogonal to each other thus it can synthetize a series of in-plane polarization states due to the field superposition with respect to different phase lags. If Δ*φ* = *π*/2 + 2*m*π (*m* is integer), it is LCP wave; if Δ*φ* = −π/2 + 2*m*π, it is RCP wave, which was intensively exploited in surface plasmon waves [34].

**Figure 1:**
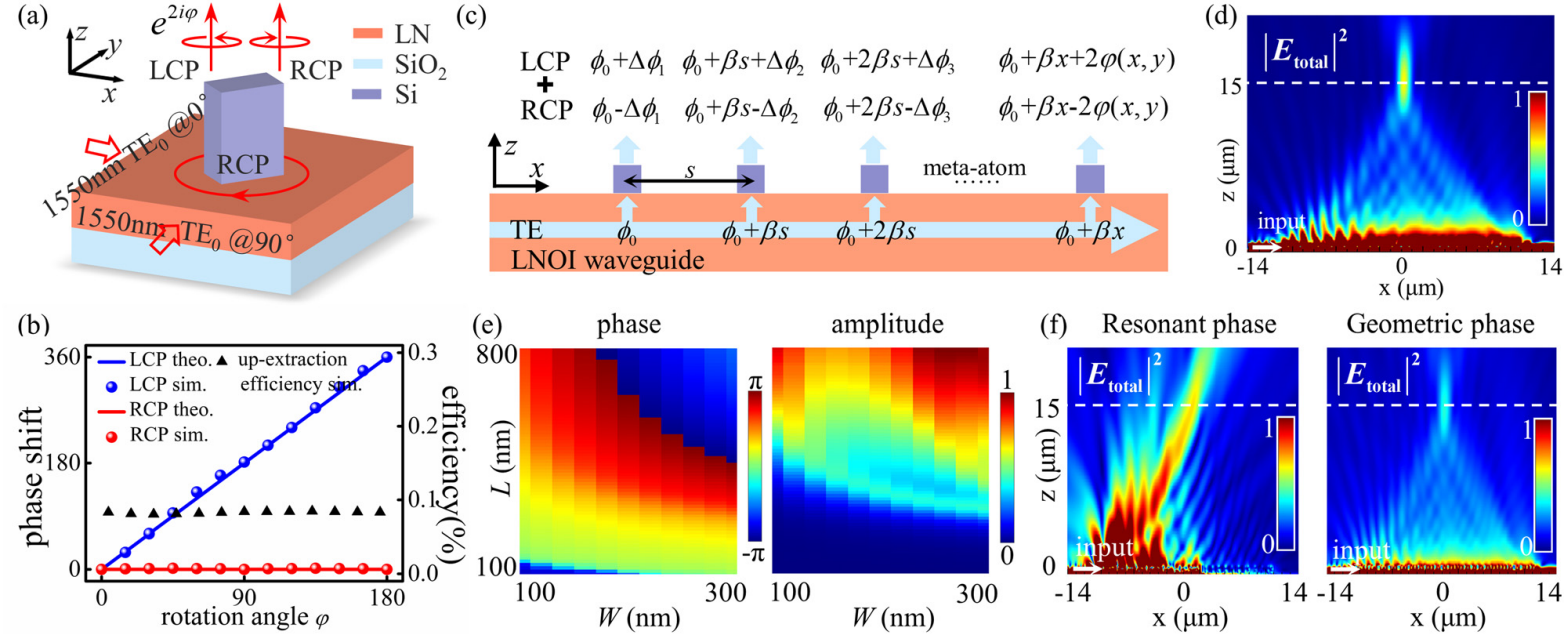
Geometric metasurface interface for guided wave manipulation. (a) Illustration of the Si metasurface integrated on an LNOI waveguide with geometric phase. (b) Numerical simulated phase shifts and up-extraction efficiency as a function of rotation angle *φ* of meta-atom in (a), driven by guide wave with synthetized RCP. (c) Working principle of geometric metasurfaces directly driven by TE eigenmode of the waveguide. The traveling TE mode can be decomposed into the combination of LCP and RCP wave, both of them are coupled out and manipulated by the geometric metasurface with different phase delays. (d) Simulation of a waveguide integrated one-dimensional metalens for off-chip light focusing, indicating the extracted RCP wave is well manipulated. The LN slab waveguide has height *H* = 300 nm, the Si antenna has length *L* = 300 nm, width *W* = 100 nm, and height *H* = 300 nm. The extracted light converged at the designed focal point (15 μm above the waveguide) at 1550-nm wavelength. (e) Simulated phase shift map and amplitude map in a parameter space of the Si meta-atom width (*W*) and length (*L*) when *H* = 600 nm, the phase shift covers 1.995π range but with very large amplitude variations. (f) Comparison of metasurfaces with resonant phase (chosen from Figure 1(e)) and geometric phase for off-chip light focusing.

Subsequently, to ensure the geometric metasurfaces driven by synthetized CP light in waveguides can work well as the free space, we perform numerical simulations by the finite difference time domain solver (FDTD solutions, Lumerical). Figure 1(a) illustrates the unit-cell structure of our simulated waveguide integrated metasurface. Here, we adopt the LNOI as the planar waveguide platform. Note that LN is an anisotropic material, and our investigation is based on *z*-cut LNOI waveguide to avoid the birefringence as light travels in the entire *x*–*y* plane. Such a capability of maintaining phase matching is favorable for integrating arbitrarily-guided structures like microrings and microdisks. The LN waveguide is designed with a thickness of 300 nm, underneath which is a 1.8-μm-thick SiO_2_ substrate. Two orthogonally propagating TE_0_ waves at 1550 nm are launched with an initial phase lag of 90° to synthetize RCP light and the amorphous Si nanorod is centrally distributed on the top, which has length *L* = 300 nm, width *W* = 100 nm, height *H* = 300 nm. The phase of the extracted light field at a few wavelengths over the waveguide and upextraction efficiency as a function of the rotation angle *φ* is illustrated in Figure 1(b). Here, the upextraction efficiency is calculated by dividing the surface integrated power flow from the surface above the metasurface structure to the input power of the waveguide. Apparently, extracted waves with the same polarization (RCP) as that of the excited wave (RCP) have no phase change (red symbol) while waves of the opposite circular polarization (LCP) obtain an additional phase change of 2*φ* (blue symbol), in good agreement with the theoretical designs (red curve and blue curve). Moreover, the scattered waves from meta-atoms with different rotation have roughly the same amplitude (black symbol), indicating an upextraction efficiency of per unitcell around 0.08%. Though the efficiency of a single unit of metasurface is low, the total efficiency is determined by summation over all units, which could be improved by increasing the number of unit cells for interaction with the guided wave since it keeps propagating in the waveguide. For such a weak perturbation condition, the nanoantenna will not disrupt the guided mode thus we can address enough numbers of antennas to manipulate the radiation of the guided wave.

Although the geometric metasurfaces driven by CP light in waveguides work efficiently, it is unpractical to use by synthesis of two beams in integrated systems. Fortunately, this geometric metasurfaces actually can be directly driven by the eigenmode of the waveguide. In analogy to linear polarization waves, a traveling TE guided wave can be decomposed into the combination of LCP and RCP wave (Figure 1(c)). For LCP wave excited metasurface, the phase distribution of the extracted LCP wave along the propagation direction (assume along the *x* direction) is *φ*_0_ + *βx*, while the phase of the extracted RCP wave is *φ*_0_ + *βx* − 2*φ*, where *φ*_0_ is the initial phase of the incidence, *βx* is the phase accumulation from the propagation and −2*φ* is the abrupt phase shift introduced by meta-atom. For RCP wave excited metasurface, the phase distribution of the extracted RCP wave is *φ*_0_ + *βx*, and the phase of the extracted LCP wave is *φ*_0_ + *βx* + 2*φ*. If arrange the meta-atoms with appropriate period (smaller than the half wavelength of guided mode), then the unwanted wave (carrying phase with *φ*_0_ + *βx*) will not be coupled out due to the phase mismatch. As a result, the rest two parts (with *βx* ± 2*φ* phase shifts) can be utilized to achieve desired free space functions. For demonstrations, we arrange the antennas along an LN slab waveguide (300 nm thick) to fulfill a one-dimensional lens phase function $\phi =2\pi /\lambda_{0}\left(f-\sqrt{x^{2}+f^{2}}\right)$ , which can focus the extracted RCP wave in free space with a focal length *f* at work wavelength *λ*_0_. The rotation angle of each antenna can be described as $\varphi =-\pi /\lambda_{0}\left(f-\sqrt{x^{2}+f^{2}}\right)+\beta x/2$ , therefore the extracted RCP wave can fulfill a convergent phase function while the extracted LCP wave is divergent. As a proof of concept, we design an integrated lens consists of 60 Si nanorods with *f* = 15 μm at 1550 nm, the center-to-center distance between adjacent nanorod is 400 nm, numerical aperture (NA) is 0.625 (we demonstrate a small lens in order to reduce the demand for computational resources). Figure 1(d) shows the simulated result driven by fundamental TE mode. Evidently, light is radiated from the waveguide and focused by the integrated metalens. Distinguished from the free space geometric metasurface designs [33], the unmodulated (residual) wave will not be coupled out to bring noise to the background, therefore such a kind of radiation beam engineering has a high signal to noise ratio (SNR = 70.06%). Here, the SNR for focusing refers to the ratio of the intensity of the focal spot (cross-sectional region of a width of 3 times the full width at half-maximum centered at the focal spot) to the total intensity on the focal plane.

In fact, there have been some reports of using guided wave driven metasurface for light manipulations based on the resonating elements [32, 35]. For comparison, we also make an investigation on resonant metasurface designs with similar condition as this geometric metasurface. Simulated abrupt phase distribution as a function of length and width of the Si nanorod (*H* = 600 nm) is displayed in Figure 1(e), which covers 1.995*π* but with very large amplitude variations. We choose meta-atom designs in Figure 1(e), whose phase shifts satisfy the phase profile and possess maximum amplitude, to construct a metalens as before. Simulated electric field distribution above the waveguide is shown in Figure 1(f). It is evident that the radiation light is heavily nonuniform in amplitude, which leads to a poor focus performance and a rapid dissipation of the energy in the waveguide. In general, the geometric metasurface has several advantages over resonant metasurface for guided wave out-of-plane manipulation. On one hand, it can easily cover 2*π* phase range by continuously rotating the nanorod without engineering and optimizing the structural parameters. On the other hand, the nanorods have entirely uniform scattering amplitudes since they are identical in geometry, which improves the performance of the devices. In addition, the phase shift of the metasurface is robust to wavelength and fabrication errors. Simulations of more meta-atom configurations are also provided in Supplementary Material Figure S1.

## 3 Function demonstrations

**Figure 2:**
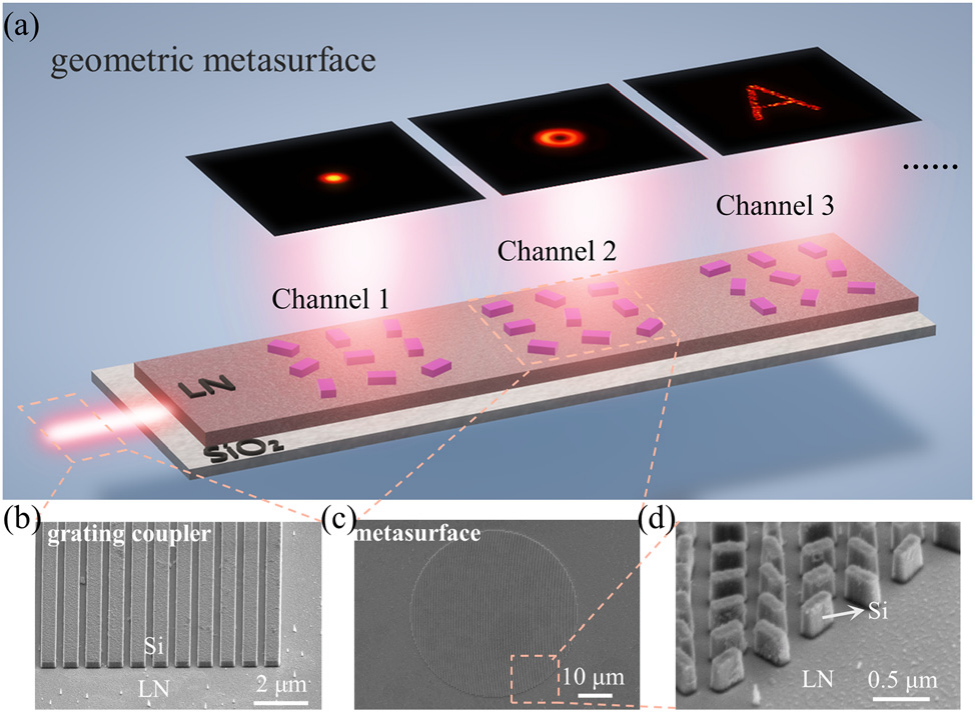
(a) Illustration of the on-chip integrated multifunctional interfaces for LNOI photonic circuit. (b) and (c) Scanning electron microscopy (SEM) images of the fabricated grating couplers and metasurface structures in our experiments. (d) Zoom-in SEM image of the details of the metasurfaces.

To show the capability of the guided wave–driven metasurfaces, we numerically and experimentally demonstrated on-chip integrated multifunctional interfaces for functional radiation waves as Figure 2(a) shows. The light is coupled into the thin film LN slab waveguide (300 nm thick) by grating couplers (Figure 2(b)) and then the guided wave is routed by waveguide on the chip. Therefore, the access waveguide can behave like a highway bus, and the metasurface arrays (Figure 2(c) and (d)) perform as different exits which convert the guided wave into different free space waves for various functions, including free-space focusing, multichannel OAM emission, holographic imaging, and so on.

### 3.1 Chip-integrated vortex beam generation and holography

Featured by its helical phase front and phase singularity, optical vortices carrying orbital angular momentum (OAM) with various topological charge *l* have proven a wide range of applications including optical communications, optical tweezers, photonic trapping, and quantum information technology [36]. Remarkably, the successful generation and manipulation of OAM modes is of great importance. Traditional attempts involve cylindrical lens mode converters, spiral phase plates, q-plates, and spatial light modulators (SLM) [37], [38], [39], [40]. Recently, integrated approaches based on fibers or silicon devices are gradually developed due to their features of small footprint and adaptation for various scenarios, including micro-ring angular grating [41], trench waveguide [42], optical vortex lattice [43], algorithm-optimized nano structures [44], holographic fork grating [45], et al.

To sufficiently validate this design principle, we propose and experimentally demonstrate an efficient chip-integrated vortex beam generator. Similarly, we consider manipulating the extracted RCP wave into a desired OAM beam meanwhile perform as a focal lens in LNOI slab waveguide (300 nm thick). The phase distribution of the focused OAM beam with *l* satisfies the following equation:

(1) $$\phi \left(x,y\right)=\frac{2\pi }{\lambda_{0}}\left(f-\sqrt{x^{2}+y^{2}+f^{2}}\right)+l\;\arctan\left(\frac{y}{x}\right)\text{,}$$

where *λ*_0_ = 1550 nm, *f* = 80 μm, the diameter of the lens *D* = 50 μm. Consequently, the rotation angle of each Si nanorod (*L* = 300 nm, *W* = 100 nm, *H* = 300 nm) can be obtained as

(2) $$\varphi =-\frac{\pi }{\lambda_{\text{0}}}\left(f-\sqrt{x^{2}+y^{2}+f^{2}}\right)-\frac{l}{2}\arctan\left(\frac{y}{x}\right)+\frac{\beta x}{2}\text{.}$$

**Figure 3:**
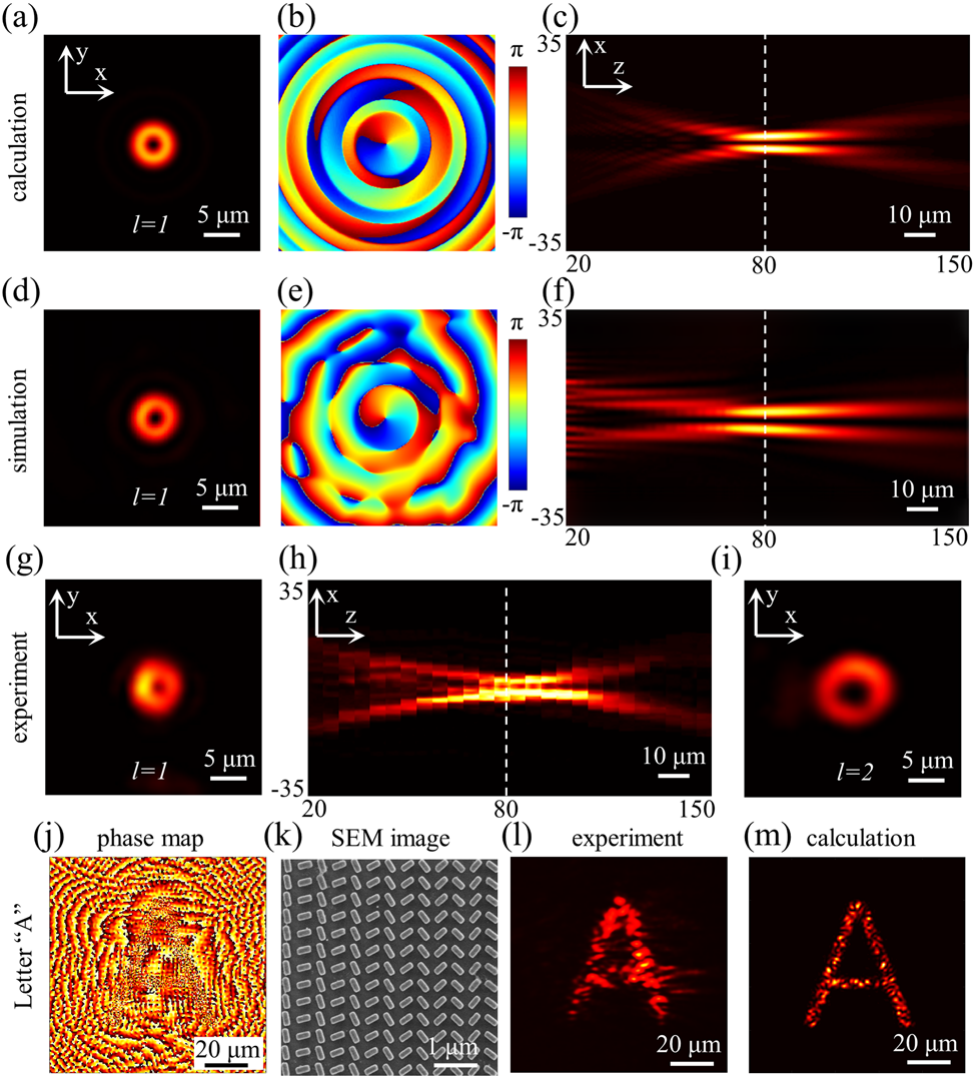
Chip integrated focused vortex beam generation (Figure 3(a)–(i)) and hologram generation (Figure 3(j)–(m)) by geometric metasurfaces based on LNOI waveguide. Theoretical calculations (*l* = 1) of (a) intensity distribution and (b) phase distribution on the focal plane, (c) intensity distribution in propagation plane. (d)–(f) Corresponding simulation results performed by FDTD solutions. (g) Experimentally measured OAM beam intensity distribution (characteristic donut shape) on the focal plane. (h) Reconstructed intensity distribution in *x*-*z* propagation plane. (i) Experimentally measured OAM beam with *l* = 2, which has a larger radius of the donut shape. (j) Calculated phase distribution of the hologram of letter “A”. (k) SEM image of part of the fabricated structures. (l) and (m) Reconstructed holographic image compared with the theoretical result.

Firstly, we demonstrate a focused OAM beam generator with *l* = 1. Based on the Huygens–Fresnel principle [46], the evolution of the OAM was calculated, in which nanostructures are considered as subsources radiating guide waves into free space with designed initial phases. The donut shape intensity profile and phase distribution on the focal plane as well as the intensity distribution in propagation plane are illustrated in Figure 3(a)–(c), indicating a focal spot size around 5 μm and *f* = 80.3 μm. Meanwhile, we perform a three-dimensional full-wave simulation by FDTD solutions (Figure 3(d)–(f)), which are in consistent with the theoretical results. Afterward, we fabricated Si metasurface and grating couplers pattered on the LNOI slab waveguide by standard electron beam lithography (EBL, ELS-F125, Elionix) and inductively coupled plasma reactive ion etching process (ICP-RIE, HSE200, Naura). The fabrication details and optical characterizations are provided in Figure S2 and S3 in Supplementary Material. A laser beam with wavelength of 1550 nm was firstly coupled into the waveguide to excite the TE mode and then scattered into the free space by the metasurfaces. Figure 3(g) shows the measured spot intensity profile of the focused OAM beam with *l* = 1 at a propagation distance of 80 μm at *λ*_0_ = 1550 nm, a characteristic dark spot with zero intensity in the center was observed. The intensity distribution at different distances above the waveguide was also measured and reconstructed in the *x–z* plane (Figure 3(h)), in good accordance with the calculation and simulation results. The wavelength response of such sample was also investigated in Supplementary Material (see Figure S4). Besides, we also fabricated other samples with different topological charges (such as *l* = 2). The corresponding experimental results are illustrated in Figure 3(i). As expected, OAM beam with larger *l* has a larger radius of the donut shape.

Furthermore, we demonstrate a chip-integrated hologram generator by our geometric metasurface designs. Based on the Gerchberg–Saxton algorithm, we calculate the phase distribution of a letter “A” on the sample hologram (Figure 3(j)). The hologram of “A” has sizes of 100 μm and produces image at a distance of 100 μm. Figure 3(k) shows the SEM image of part structures of the fabricated meta-hologram. The experimental image reconstructed from the hologram under illumination with a TE mode of 1550 nm is shown in Figure 3(l), agreeing well with the calculated result (Figure 3(m)). The quality degradation in experiment may attribute to the nonidentical extraction of each structure, since the sample spans a long propagation distance and the energy dissipation of the guided mode will be taken into account. Besides, a Gaussian beam was employed for illumination to excite the guided wave, which definitely deviates from uniform plane wave intensity.

### 3.2 Multichannel vortex beam generation

Although we have successfully generated different OAM beams assisted with different geometric metasurfaces, each sample can only produce one OAM beam with single topological charge, which will not be able to meet the requirements in many scenarios. Especially in applications of information processing and optical communications, different types of OAM beams are often needed at the same time. As a consequence, we demonstrate integrated multichannel vortex beam generation with two different methods based on the LNOI slab waveguide.

#### 3.2.1 Multiplexing by holographic design

To endow a singlet metasurface with the capability to generate multichannel OAM beams, we designed the complex field of the OAM beams in the metasurfae plane (*z* = 0), which can be expressed as the linear superposition of the complex field of each beam:

(3) $$\begin{array}{c} E\left(x,y,0\right)=\sum_{n=1}^{N}a_{n}\left(x,y,0\right)\exp\left(-i\left(l\theta \left(x,y\right)+\varphi_{rand}\right)\right)\\ \exp\left(-i\left(k_{0}\;\sin\;\theta_{nx}x+k_{0}\;\sin\;\theta_{ny}y\right)\right), \end{array}$$

where *E*(*x*, *y*, 0) is the synthetic complex electric field of all channels at the structure plane, *N* is the total number of channels which is limited to the size of the sample. *a*_*n*_(*x*, *y*, 0) is the amplitude distribution of individual OAM beam at the metasurface plane, which is assumed as a constant in our design to ensure equal energy for each channel and simplify the calculation. *θ*(*x*, *y*) = arctan(*y*/*x*) is the azimuthal angle, *l* is the topological charge of each beam, *k*_0_ = 2*π*/*λ*_0_ is the wavenumber in the free space, *θ*_*nx*_ and *θ*_*ny*_ are the propagation angles of the light with respect to the *x* and *y* directions, respectively. Additionally, to minimize the mutual interference of adjacent channels, a random phase *φ*_rand_ is introduced. Then the amplitude and phase distribution in the metasurface plane can be deduced as

(4) A(x,y,0)=abs(E(x,y,0)),

(5) ϕ(x,y,0)=angle(E(x,y,0)).

For simplicity, we utilize a phase-only hologram to reconstruct the OAM beams and ignore the amplitude distribution. At the same time, a lens phase profile is also added to manipulate each OAM beam focusing. Therefore the final phase profile provided by the meta-atoms is presented as

(6) $$\phi_{all}\left(x,y,0\right)=\frac{2\pi }{\lambda_{0}}\left(f-\sqrt{x^{2}+y^{2}+f^{2}}\right)+\phi \left(x,y,0\right).$$

**Figure 4:**
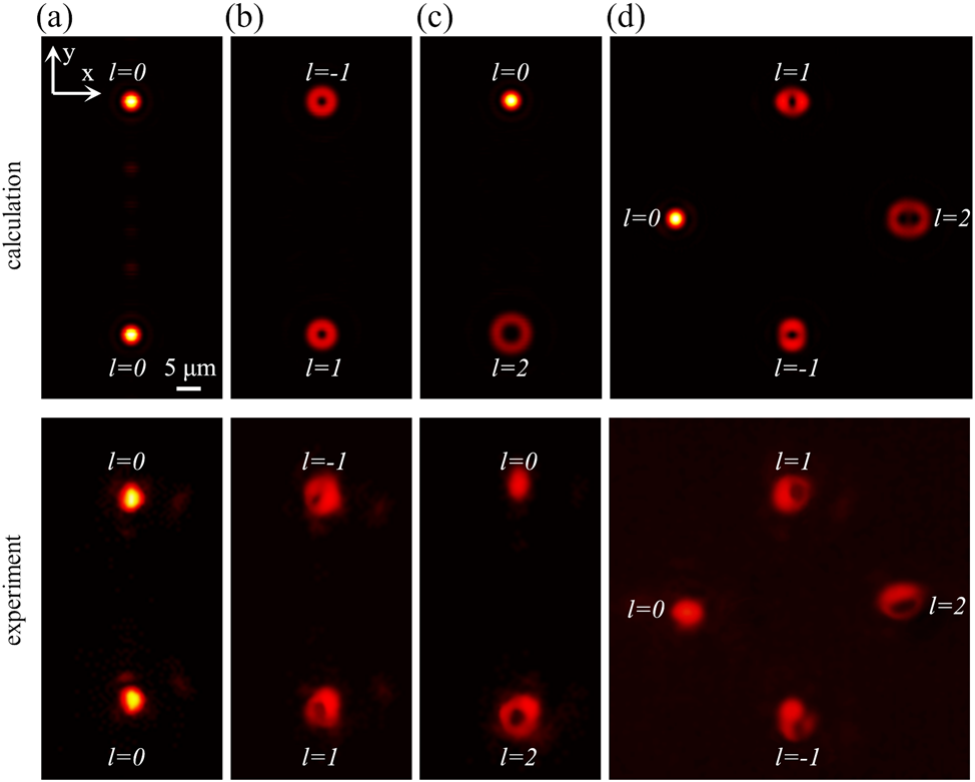
Theoretical calculations and experimental results of the focused multi-channel OAM beams achieved by holography multiplexing. Two-channel beams with (a) *l* = 0 and 0, (b) *l* = −1 and 1, (c) *l* = 0 and 2. (d) Four-channel beams with *l* = −1, 0, 1, 2. All of them have the same scale bar.

Afterward, we demonstrate four different multichannel beam generators with focal plane of *f* = 80 μm at *λ*_0_ = 1550 nm. Figure 4(a)–(d) show the calculated (top row) and measured (bottom row) intensity distributions of the double-channel beams and four-channel beams at the focal plane, respectively. In our design, each multiplexed metasurface is a circular region with a diameter of 50 µm and the period of the unit cell is 400 nm.

#### 3.2.2 Cascaded emitter array

**Figure 5:**
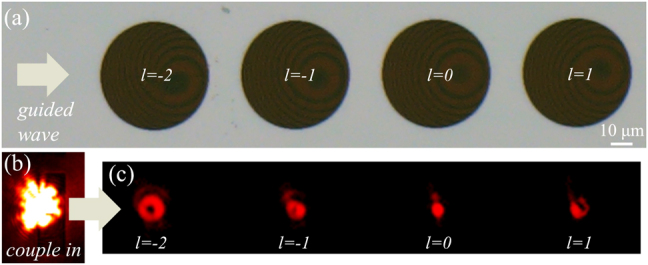
Experimental demonstration of the cascaded focused OAM emitter array. (a) Optical microscopic image of the fabricated array consists of four emitters with different *l* = −2, −1, 0, 1. In experiments, TE_0_ guided wave is excited and propagates from left to right. (b) Illumination onto the couple-in grating at *λ*_0_ = 1550 nm. (c) Simultaneously measured intensity patterns on the focal plane emitted from the array. The imperfections may be due to the diffraction effect inside the waveguide.

In addition to multiplexing based on holography, we also utilize metasurface array to achieve multi-channel beam output. Benefited from the low propagation loss of the guided wave (less than 1 dB/cm by estimation) and the small energy consumption of a singlet metasurface radiation, it is feasible to extract the light in the same access waveguide for several times by cascaded multiple metasurface exits. We experimentally fabricated an OAM emitter array consisting of four different metasurfaces (*D* = 50 μm, *f* = 80 μm) distributed on the same LNOI slab waveguide (Figure 5(a)) and simultaneous emission of different vortices (*l* = −2, −1, 0, 1) has been verified in Figure 5(c). The quality of the manipulated beam declines slightly at a longer propagation distance may be caused by the diffraction effect of light in slab waveguide, which can be improved by careful optimizations.

## 4 Discussion and conclusion

Honestly speaking, the current design used identical meta-atoms for manipulation by PB mechanism, the scattering efficiencies actually differ slightly everywhere due to the propagation/extraction loss of the waveguide mode. But in our demonstration, the amplitude variations are negligible, since we implemented a weak scattering process within a short interaction distance. A feasible solution to rigorously balance the extraction efficiency is to incorporate geometric phase with resonant mechanism for manipulation of phase together with amplitude. In this way, we are able to construct uniform-intensity extraction or arbitrary extraction intensity profiles. Moreover, benefited from the excellent electro-optic property of LN in combination with metasurface interfaces, chip integrated dynamic devices for optical communications and displays could be expected, ranging from optical phase arrays to tunable OAM beam generators, as well as miniaturized display devices for virtual reality and augmented reality.

In summary, we have proposed a robust interface of subwavelength geometric metasurface integrated to LNOI waveguide, providing a convenient and highly versatile platform for bridging a link between guided waves and free-space functional beams. Assisted with geometric phase metasurface, we experimentally demonstrated off-chip beam focusing, OAM generations, and holography in LNOI slab waveguides. By holographic multiplexing and metasurface array design, we also performed multichannel OAM emissions. Our developed technology will pave exciting ways for full control of light across integrated photonics and free-space platforms, promising a plethora of multifunctional LN devices.

## Supplementary Material

Supplementary Material

## References

[1] Arizmendi L. (2004). Photonic applications of lithium niobate crystals. *Phys. Status Solidi A*.

[2] Nikogosyan D. N. (2005). *Nonlinear Optical Crystals: A Complete Survey*.

[3] Boes A., Corcoran B., Chang L., Bowers J., Mitchell A. (2018). Status and potential of lithium niobate on insulator (LNOI) for photonic integrated circuits. *Laser Photon. Rev.*.

[4] Qi Y., Li Y. (2020). Integrated lithium niobate photonics. *Nanophotonics*.

[5] Lin J., Bo F., Cheng Y., Xu J. (2020). Advances in on-chip photonic devices based on lithium niobate on insulator. *Photon. Res.*.

[6] Wang C., Zhang M., Chen X. (2018). Integrated lithium niobate electro-optic modulators operating at CMOScompatible voltages. *Nature*.

[7] Pohl D., Escalé M. R., Madi M. (2019). An integrated broadband spectrometer on thinfilm lithium niobate. *Nat. Photonics*.

[8] Zhang M., Buscaino B., Wang C. (2019). Broadband electro-optic frequency comb generation in a lithium niobate microring resonator. *Nature*.

[9] Fang B., Gao S., Wang Z., Zhu S., Li T. (2021). Efficient second harmonic generation in silicon covered lithium niobate waveguides. *Chin. Opt. Lett.*.

[10] Krasnokutska I., Chapman R. J., Tambasco J. J., Peruzzo A. (2019). High coupling efficiency grating couplers on lithium niobate on insulator. *Opt. Express*.

[11] He L., Zhang M., Shams-Ansari A., Zhu R., Wang C., Marko L. (2019). Low-loss fiber-to-chip interface for lithium niobate photonic integrated circuits. *Opt. Lett.*.

[12] Van Acoleyen K., Bogaerts W., Jágerská J., Le Thomas N., Houdré R., Baets R. (2009). Off-chip beam steering with a one-dimensional optical phased array on silicon-on-insulator. *Opt. Lett.*.

[13] Van Acoleyen K., Komorowska K., Bogaerts W., Baets R. (2011). One-dimensional off-chip beam steering and shaping using optical phased arrays on silicon-on-insulator. *J. Lightwave Technol.*.

[14] Sun J., Timurdogan E., Yaacobi A., Hosseini E. S., Watts M. R. (2013). Large-scale nanophotonic phased array. *Nature*.

[15] Yu N., Capasso F. (2014). Flat optics with designer metasurfaces. *Nat. Mater.*.

[16] Genevet P., Capasso F., Aieta F., Khorasaninejad M., Devlin R. (2017). Recent advances in planar optics: from plasmonic to dielectric metasurfaces. *Optica*.

[17] He Q., Sun S., Xiao S., Zhou L. (2018). High-efficiency metasurfaces: principles, realizations, and applications. *Adv. Opt. Mater.*.

[18] Li Z., Yu S., Zheng G. (2020). Advances in exploiting the degrees of freedom in nanostructured etasurface design: from 1 to 3 to more. *Nanophotonics*.

[19] Yu N., Genevet P., Kats M. A. (2011). Light propagation with phase discontinuities: generalized laws of reflection and refraction. *Science*.

[20] Chen C., Song W., Chen J.-W. (2019). Spectral tomographic imaging with aplanatic metalens. *Light Sci. Appl.*.

[21] Wang L., Kruk S., Tang H. (2016). Grayscale transparent metasurface holograms. *Optica*.

[22] Xu B., Li H., Gao S. (2020). Metalens-integrated compact imaging devices for wide-field microscopy. *Adv. Photon.*.

[23] Meng Y., Liu Z., Xie Z. (2020). Versatile on-chip light coupling and (de)multiplexing from arbitrary polarizations to controlled waveguide modes using an integrated dielectric metasurface. *Photon. Res.*.

[24] Zhang Y., Li Z., Liu W. (2019). Spin-selective and wavelength-selective demultiplexing based on waveguide-integrated all-dielectric metasurfaces. *Adv. Opt. Mater.*.

[25] Xie Z., Lei T., Qiu H., Zhang Z., Wang H., Yuan X. (2020). Broadband on-chip photonic spin Hall element via inverse design. *Photon. Res.*.

[26] Shen B., Wang P., Polson R., Menon R. (2015). “An integrated-nanophotonics polarization beamsplitter with 2.4 × 2.4 μm^2^ footprint. *Nat. Photonics*.

[27] Wang K., Ren X., Chang W., Lu L., Liu D., Zhang M. (2020). Inverse design of digital nanophotonic devices using the adjoint method. *Photon. Res.*.

[28] Wang H., Zhang Y., He Y., Zhu Q., Sun L., Su Y. (2019). Compact silicon waveguide mode converter employing dielectric metasurface structure. *Adv. Opt. Mater.*.

[29] Li Z., Kim M. H., Wang C. (2017). Controlling propagation and coupling of waveguide modes using phase-gradient metasurfaces. *Nat. Nanotechnol.*.

[30] Wang C., Li Z., Kim M. H. (2017). Metasurface-assisted phase-matching-free second harmonic generation in lithium niobate waveguides. *Nat. Commun.*.

[31] Fang B., Li H., Zhu S., Li T. (2020). Second-harmonic generation and manipulation in lithium niobate slab waveguides by grating metasurfaces. *Photon. Res.*.

[32] Guo X., Ding Y., Chen X., Duan Y., Ni X. (2020). Molding free-space light with guided wave-driven metasurfaces. *Sci. Adv.*.

[33] Huang L., Chen X., Mühlenbernd H. (2012). Dispersionless phase discontinuities for controlling light propagation. *Nano Lett.*.

[34] Li L., Li T., Tang X. M., Wang S. M., Wang Q. J., Zhu S. N. (2015). Plasmonic polarization generator in well-routed beaming. *Light Sci. Appl.*.

[35] Xie C., Huang L., Liu W. (2021). Bifocal focusing and polarization demultiplexing by a guided wave-driven metasurface. *Opt. Express.*.

[36] Shen Y., Wang X., Xie Z. (2019). Optical vortices 30 years on: OAM manipulation from topological charge to multiple singularities. *Light Sci. Appl.*.

[37] Beijersbergen M. W., Allen L., Vanderveen H., Woerdman J. P. (1993). Astigmatic laser mode converters and transfer of orbital angular momentum. *Opt. Commun.*.

[38] Beijersbergen M. W., Coerwinkel R. P. C., Kristensen M., Woerdman J. P. (1994). Helical-wave front laser beams produced with a spiral phase plate. *Opt. Commun.*.

[39] Slussarenko S., Murauski A., Du T., Chigrinov V., Marrucci L., Santamato E. (2011). Tunable liquid crystal qplates with arbitrary topological charge. *Opt. Express*.

[40] Forbes A., Dudley A., McLaren M. (2016). Creation and detection of optical modes with spatial light modulators. *Adv. Opt Photon*.

[41] Cai X., Wang J., Strain M. J. (2012). Integrated compact optical vortex beam emitters. *Science*.

[42] Zheng S., Wang J. (2017). On-chip orbital angular momentum modes generator and (de)multiplexer based on trench silicon waveguides. *Opt. Express*.

[43] Du J., Wang J. (2017). Chip-scale optical vortex lattice generator on a silicon platform. *Opt. Lett.*.

[44] Xie Z., Lei T., Li F. (2018). Ultra-broadband on-chip twisted light emitter for optical communications. *Light Sci. Appl.*.

[45] Zhou N., Zheng S., Cao X. (2019). Ultra-compact broadband polarization diversity orbital angular momentum generator with 3.6 × 3.6 um^2^ footprint. *Sci. Adv.*.

[46] Li L., Li T., Wang S. M., Zhang C., Zhu S. N. (2011). Plasmonic airy beam generated by in-plane diffraction. *Phys. Rev. Lett.*.

