# Supplementary material for: Manipulating guided wave radiation with integrated geometric metasurface
Source: Nanophotonics. 2021 Oct 13;11(9):1923–30. doi: 10.1515/nanoph-2021-0466 (PMC11501629; doi:10.1515/nanoph-2021-0466)
Supplement: Supplementary file 1 — Supplementary Material [file j_nanoph-2021-0466_suppl.docx]

**Supplementary Information for**

**Manipulating guided wave radiation with integrated geometric metasurface**

*Bin Fang^1,2,3^, Zhizhang Wang ^1,2^, Shenglun Gao ^1,2^, Shining Zhu^1,2^, Tao Li^1,2*^*

^1^National Laboratory of Solid State Microstructures, Key Laboratory of Intelligent Optical Sensing and Manipulation, Jiangsu Key Laboratory of Artificial Functional Materials, College of Engineering and Applied Sciences, Nanjing University, Nanjing, 210093, China.

^2^Collaborative Innovation Center of Advanced Microstructures, Nanjing, 210093, China

^3^College of Optical and Electronic Technology, China Jiliang University, Hangzhou, 310018, China

*Corresponding author: [taoli@nju.edu.cn](mailto:taoli@nju.edu.cn)

**Supplementary Appendix A: Comparison of different meta-atom configurations**

Although various materials can be employed for defining the meta-atom loaded on the LNOI waveguide, we choose *α*-Si as the ideal material for a few reasons. First, silicon has a pretty high refractive index contrast compared with LN, benefiting for light extraction and manipulation with high efficiency from the waveguide. Second, the nanofabrication technology for silicon is mature and has been well-developed for integrated photonics and nanophotonics. Moreover, it also has a rather low optical absorption loss and has been demonstrated for plentiful high-quality devices at telecom wavelengths. Furthermore, the combination of silicon and LN may promise a desirable platform for emerging hybrid integrated devices and applications.

Actually, we also have made an investigation on different materials of the meta-atom for waveguide integrated metalens designs by geometric phase. Several simulated results are exhibited in Figures S1(a)-S1(f), including Si, SiN_x_, LN, TiO_2_, Au and etched hole (the optical properties of the materials in the simulation are extracted from our experimental data and Palik database). It can be concluded that a larger refractive index perturbation introduced by metasurfaces corresponds to a stronger extracted energy from the waveguide and a faster decay of energy of the mode. Though plasmonic metasurface can enhance the radiation of the guided wave, it is accompanied with high ohmic loss for energy waste. In this regard, the dielectric silicon is an optimal choice currently considering the good performance and compatible to Silicon photonics platform.


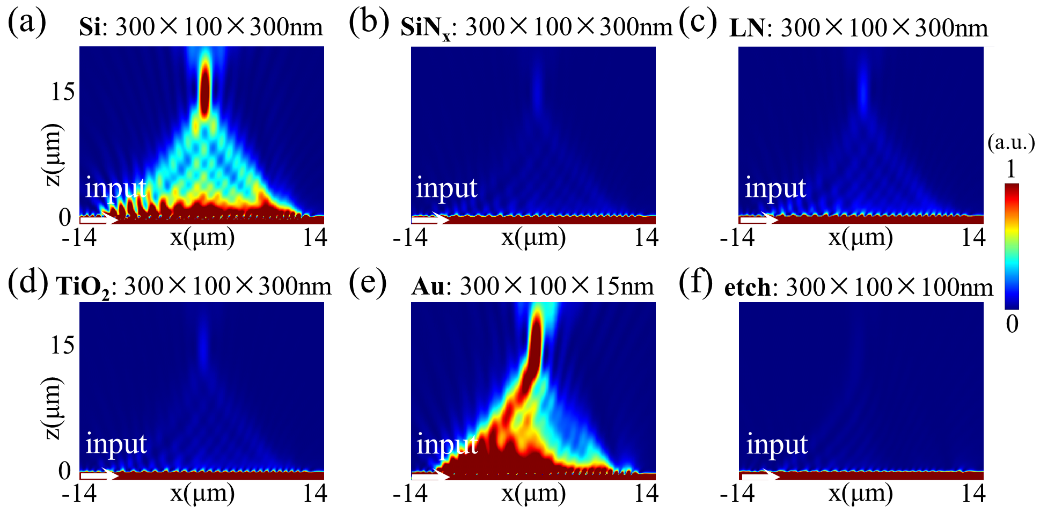


**Figure S1.** Simulated results of the metalens integrated on a LNOI waveguide (300 nm thick and 1 μm width) with (a) Si metasurface, (b) SiN_x_ metasurface, (c) LN metasurface, (d) TiO_2_ metasurface, (e) Au metasurface and (f) etched hole metasurface. The size of the unit cell (length × width × height) is labeled on the top and the period is 400nm.

**Supplementary Appendix B: Sample fabrication**

A commercially available LNOI wafer (available in NANOLN Company) with 300-nm-thick LN device layer and 1.8-μm buried silicon dioxide was firstly cleaned by sonication in acetone and dried with a N_2_ stream. A 300-nm-thick *α*-Si film was then deposited on top surface of the LNOI thin film by plasma-enhanced chemical vapor deposition (PECVD). Subsequently, positive electron beam resist (PMMA) was spin-coated and followed by EBL process (ELS-F125, Elionix) to define the metasurface and coupler patterns. After the development, a 30-nm thick chromium (Cr) film was deposited onto the wafer using electron-beam evaporation, followed by overnight lift-off to transfer the patterns. Then the patterned chromium layer served as a hard mask for dry etching the Si film in a mixture of C_4_F_8_ and SF_6_ plasma (HSE200, Naura). Finally, the remaining chromium mask was removed by ammonium cerium nitrate (Figure S2).


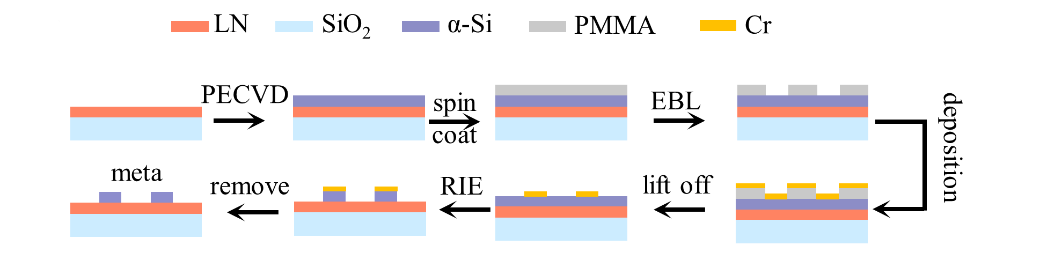


**Figure S2.** Fabrication sequence of metasurfaces and couplers patterned on the LNOI slab waveguide. Standard electron beam lithography and reactive ion etching technology was employed.

**Supplementary Appendix C: Experimental measurements**

The optical setup used for characterizing our guided wave–driven metasurfaces is shown in Figure S3. A continuous-wave tunable laser (Fianium Super-continuum) was employed for illumination from the substrate side of the sample. The free-space beam output from the laser firstly passed through a linear polarizer and was then focused onto the grating couplers with an objective (100× magnification, NA = 0.7) to excite the TE mode of the slab waveguide. Afterwards, on the other side, the light scattered into free space by metasurfaces on top of the LN waveguide was collected by another objective (50× magnification, NA = 0.42) and then projected into a near-infrared camera (Xenics Xeva 1083) for imaging.


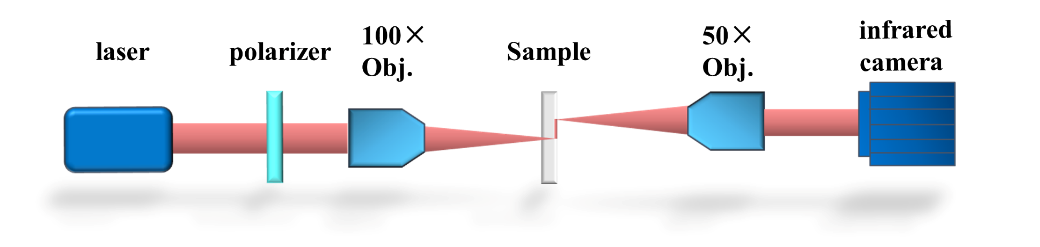


**Figure S3.** Schematic of the experimental setup for measurement. A linear polarized light is incident to excite the TE_0_ mode of the waveguide. The scattered intensity distribution by the metasurfaces at different distances can be detected by the near-infrared camera without polarization analyzer.

**Supplementary Appendix D: Broadband property of integrated metasurface**

In the experiment, we measured the generation of the focused vortex beam (*l* = 1) for different incident wavelengths and clearly observed the characteristic beam profile for all wavelengths on the same focal plane (Figure S4). In principle, the scattered local phase is a sum of phase accumulation from the propagation and abrupt phase delay induced by meta-atom. Though phase provided by the geometric metasurface is wavelength independent, the dispersion effects of the propagation phase in waveguides still exist. As a result, the beam spot will shift towards the input port as the wavelength increases. Besides, the diffraction dispersion of the lens phase profile will also have an impact on the distortion of the spot.


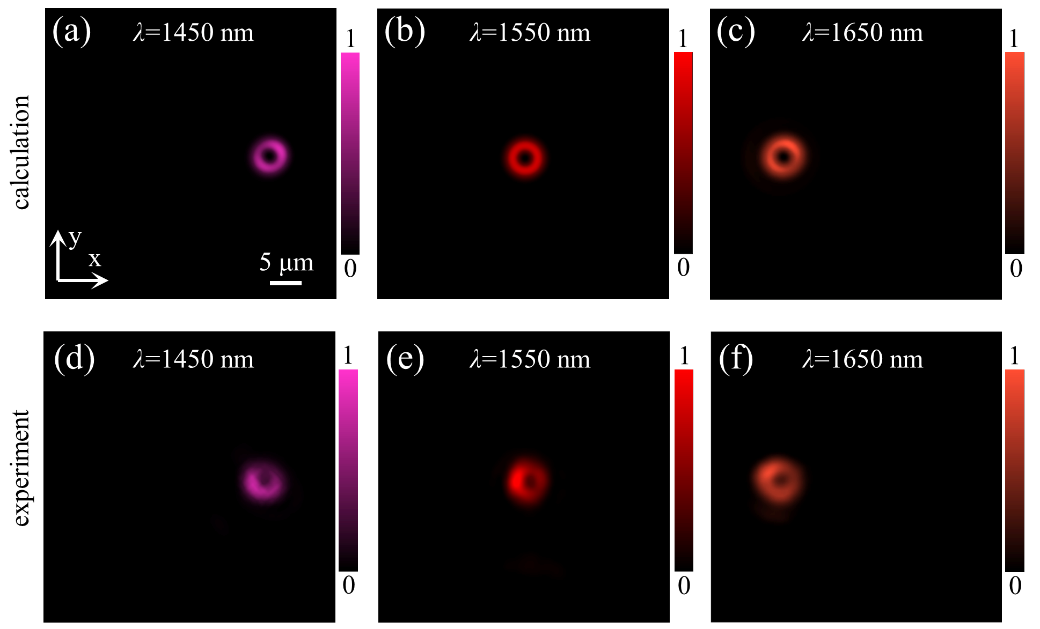


**Figure S4.** Broadband property of integrated geometric metasurfaces for OAM generation. Calculated and measured spots on the focal plane (*z* = 80 μm) of (a) and (d) *λ* = 1450 nm, (b) and (e) *λ* = 1550 nm, (c) and (f) *λ* = 1650 nm. The displacement of the focal spot along the *x*-axis and the distortion is attributed to the dispersion effects of the propagation phase in waveguides and the diffraction dispersion of the lens phase profile, while the phase provided by the geometric metasurface is wavelength independent. Here the pseudocolor is a guide to the eye for visualizing the different wavelengths.

In our experimental system, the free space light was firstly coupled into the slab waveguide by a grating coupler while its coupling efficiency differs greatly for different wavelength. Therefore, it is not suitable to directly characterize the metasurfaces’ efficiency performance as a function of wavelength. Instead, we provide a simulated result of different wavelength (Figure S5) to help evaluate the efficiency of metasurface. We demonstrate a small OAM generator (the diameter of the sample *D* = 8 μm, *f* = 4 μm) in order to reduce the demand for computational resources. Figure S5 (a)-(c) show the intensity distribution on the focal plane, all of which share the same color bar. In the simulation, the input power for different wavelength in the waveguide keeps the same, so it is fair to compare their up-extraction efficiency performance. By calculation, the total up-extraction efficiency for three wavelength is about 1.09%, 1.6%, 1.16%, respectively. Considering the diameter of the sample in experiments *D* = 50 μm, the total up-extraction efficiency can be roughly estimated around 6.8%, 10%, 7.25%.


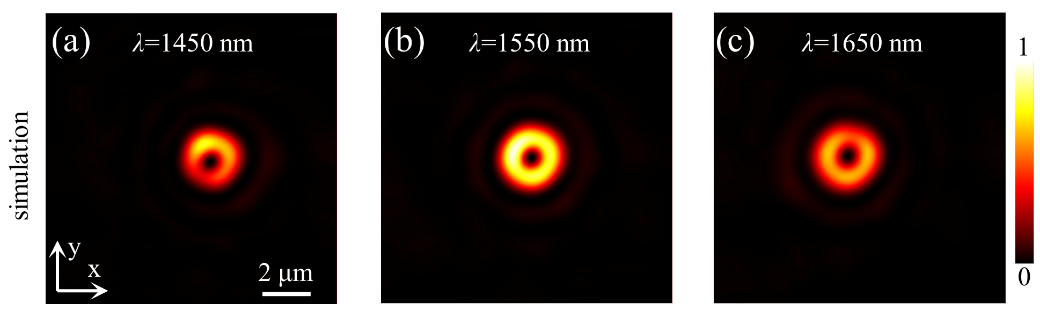


**Figure S5.** Simulated results to help evaluate the total efficiency of metasurface. The intensity distribution of the OAM beam on the focal plane of (a) λ = 1450 nm, (b) λ = 1550 nm, (c) λ = 1650 nm, all of which share the same color bar. In the simulation, the input power for different wavelength in the waveguide keeps the same.
